# The British Hospitals Association

**Published:** 1911-10-14

**Authors:** 


### THE BRITISH HOSPITALS ASSOCIATION.

### Manchester Conferences.

### MEDICAL STAFF AND HOSPITAL BOARDS: THE DISTRIBUTION OF SATURDAY AND SUNDAY FUNDS.

As The Hospital stated last Saturday, the discussion of the British Hospitals Association on the Insurance Bill was prolonged to the second day. After the resolution had been carried, the business of the Conference was resumed with the reading of a paper on

"THE REPRESENTATION OF HONORARY MEDICAL STAFFS UPON BOARDS OF MANAGEMENT OF VOLUNTARY HOSPITALS," by D. E. S. Reynolds, Chairman of the Medical Board of Manchester Royal Infirmary.

Some ten years ago, owing to an acute disagreement between the lay and medical elements of one of the London hospitals, a series of articles appeared in the *Practitioner* (Vol. 13, 1901), on the question of hospital management from the lay and medical points of view. The arguments in favour of medical representation on Boards of Management are so convincing that further discussion on the subject seems to me to savour of the profitless undertaking of flogging a dead horse; and I was unwilling to undertake the request that I should read this paper until I learned, with surprise, that there are still hospitals in this country in which such arrangements do not exist.

To shorten my argument, I take it for granted that every hospital possesses a lay Board of Management (composed entirely, or almost entirely, of laymen) and a separate Medical Board (composed of the honorary medical and surgical staff), and these I will allude to below as "the Board" and "the Staff," respectively.

Now it is always well in dealing with an adversary in argument to consider as calmly as possible why it is he can persist in being so adamant as not at once to alter his opinion in accordance with the views you put before him, and so far as I can surmise, the reasons for a purely lay administration of hospitals are, (*a*) the question of ownership; (*b*) the finding and spending of money; (*c*) business capacity; (*d*) possible censure of one or more members of the honorary staff; (*e*) lay suspicion of medical hospital practice.

To consider these seriatim: (*a*) *Ownership*.—I suppose that in law a voluntary hospital is owned by the trustees, who are entirely, or almost entirely, laymen, and it naturally seems to follow that they should manage their own property. This highly complex organism, a hospital, comprises not only buildings and paid lay and medical officers, but also, as a most important and indispensable part of its working units, an honorary medical staff; and just as the paid officers have naturally no voice in the management, so it is supposed that the honorary staff should have no voice. But it should be remembered that a large number of hospitals in this country have been initiated by medical men in the district, partly from philanthropic motives, and partly, to put it broadly, but in no sinister sense, for purposes of self-education and advertisement; and it seems somewhat arbitrary that, having once started the hospital, they or their successors should take no further part in the management. Secondly, the honorary staff are not paid except indirectly by the experience gained and the advertisement of being on the staff; and if, owing to bad management, the hospital is a failure, the reputation of the staff may suffer enormously.

(*b*) The obtaining of money to maintain a hospital is largely the work of the Board, but this does not seem to me to be a reason why the staff should not have a voice in the expenditure. I am sure the Chancellor of the Exchequer could not well object to the First Lord of the Admiralty and other members of the Cabinet having a say in the Naval Estimates. Moreover, it is by no means unknown for members of the honorary staff not only to contribute to the funds of the hospital, but also to obtain large sums from their richer patients.

(*c*) It is frequently stated, and in the articles in the *Practitioner* already alluded to is rather insisted on, that members of the medical profession are very lacking in business capacity. I admit that some of them are, but I have seldom known a successful medical man who was not businesslike in his habits. Want of business capacity is a fault which has been commonly associated for centuries with scientists and artists, and has frequently been made the subject of mirth; and in spite of this, such unbusinesslike people have won the greatest renown, often indeed, however, without making great fortunes; but taking the average run of successful doctors they are, in my experience, quite as businesslike as other members of the community. I have even known more than one unbusinesslike lawyer.

(*d*) It may be urged that if it is deemed necessary to censure a member of the honorary staff, either for irregular attendance or irregular conduct, it would be difficult to do so if the member himself or one of his colleagues is sitting on the Board. But, firstly, such occasions are very rare; and secondly, it is exactly on such occasions that mistakes may be made by laymen which a word from a member of the staff will rectify at once, and so prevent possible friction and misunderstandings.

(*e*) Finally, it may be argued that the staff should not be represented on the Board because of the suspicion which many of the lay public unfortunately have about medical work in hospitals, and especially in hospitals to which medical schools are attached. The idea is that "the doctors must be looked after." I do not believe there is the slightest foundation for this suspicion; but knowing that it exists, I think it is in the highest degree desirable that hospitals should be managed by laymen, who should be in overwhelming preponderance in number on the Board; but this is no reason why some of the Board should not be drawn from the staff, rather, indeed, that they should be present, so that they may hear at first hand, and be able to correct at once unjust allegations made against them or their colleagues.

The above are the only reasons I can think of for the exclusion of staff representatives from Boards of Management; possibly other reasons may be brought forward by the members of this Conference.

Let us now consider reasons why the staff should be represented on the Board. It is obvious that some means must exist by which the views and requests of the Board and of the staff can be communicated one to the other. The three methods of intercommunication are by resolutions placed on the minute book or forwarded by letter, conference, or by direct representation of members of the staff on the Board. (A point arises here which may be at once disposed of; it may be argued that if the staff are represented on the Board of Management, the latter should be represented at the meetings of the staff. But as a matter of experience, the meetings of the staff are taken up almost entirely with purely medical or teaching matters, and also—at any rate at the Manchester Royal Infirmary—in the rare event of the Board wishing to discuss matters with the full staff, a conference is always most readily arranged.)

In the method by resolution, or by letter, the communication may be ignored by the other parties; it may be misunderstood, or not clearly and fully worded, and may thus require a whole mass of correspondence, thus causing a delay of weeks or months over a matter which a personal explanation would settle in a few minutes. The method by conference is better, but again involves unnecessary delay in arranging a time and place to suit all parties. By regular representation these delays and misunderstandings are at once done away with. Misunderstandings may and do arise, first because of the different points of view from which lay and medical men look at certain questions, and secondly, because of the somewhat extraordinary ignorance sometimes displayed by lay members of Hospital Boards about hospital matters, and not infrequently even on matters relating to the very hospital on whose Board they serve; this ignorance may be found more particularly in those members who may possibly attend quite regularly, but who are very busy public men, who have already too many irons in the fire. Examples of misunderstandings, because of a different view-point, may arise on certain subjects which are principally professional but which the layman sometimes thinks his knowledge is of great importance, such subjects as relate to the decoration, furnishing, ventilation, warming, and alteration of wards; the expense or utility of certain dietaries; the use of alcohol, and the amount ordered; the necessity or otherwise for special medical and surgical appliances or methods of treatment proposed by the staff, which some layman may consider to be a mere fad, or some method proposed by the layman which the staff know to be a fad; and last, but by no means least, letters which sometimes come, even in the best regulated institutions, complaining of the treatment which patients have received. By direct representation also matters which have been dealt with by the Medical Board and entered briefly on their minutes can be at once, if necessary, explained in full detail before the Lay Board. But in many other subjects which arise at the meetings of the Lay Board, such as those relating to pure finance, many legal matters, the management of servants (other than medical), the placing of contracts, and similar purely business affairs, the layman is, and ought to be, paramount, and such matters are better left alone by the staff even if present at the Board meetings.

I have worked at hospitals under Lay Boards both with representatives of the staff and without representatives. I was resident medical officer (and as such was also secretary of the Medical Committee) of the Manchester Royal Infirmary for the four years 1887 to 1890, and during this time I remember many differences of opinion between the Board and the staff, some of these being of a somewhat acute nature; and at times each body, anxious to maintain its dignity, was as far apart from the other as the Lords and the Commons have lately been. This was a bad thing for the constitution of the hospital, but I was so used to this that I hardly knew there was a more perfect way. This I learned when, as medical officer and visiting physician to the Manchester Workhouse Infirmary, I was asked by the Board of Guardians to attend regularly at its fortnightly meetings. I then found how easy it was to straighten out in a few words what might otherwise, but for my presence, have been very crooked matters, and how, as a result, for the sixteen years I was an officer of the Poor Law and in charge of a hospital of nearly 2,000 beds, practically no friction occurred, and certainly none of a lasting character.

About eleven years ago the Board of Management of the Manchester Royal Infirmary arranged to have representatives of the staff to sit with them, and as one of the physicians I have seen with the greatest pleasure the enormous improvement in the relationship between the Board and the staff as compared with what I formerly knew, so that since this change I have never known any friction whatever to occur and the utmost good feeling exists between the various members of the Board and the staff. The system arranged seems to me to be so admirable that perhaps I may be permitted to describe it in a little more detail. The Board of Management consists of certain lay trustees, twenty-one in number, elected by the general body of trustees at their annual meeting. This Board meets once a month; exactly the same gentlemen meet once a week as a House Committee. The Medical Committee consists of all the members of the honorary staff, twenty-three in number, and meets regularly once a month to discuss any matters relating to the treatment and well-being of the patients, the diet sheets, surgical instruments, the sanitary state of the wards, the consideration of cases which have been over three months in the hospital, and all questions relating to the teaching of students. At the January meeting each year the Medical Committee elects by ballot four of its members to represent them on the Board of Management. As a rule, and it ought to be a rule, two of the elected members should be physicians and two should be surgeons, one of the four, however, being from time to time chosen from the honorary officers in charge of special departments, such as those of gynæcology, eye or ear diseases, and as a rule, and again it ought to be a rule, no member should serve as a representative for more than two years in succession, for it is most important that no one member of the staff should obtain an undue influence on the Board. These four elected members now become full members for the year of the Board of Management, and are summoned by the General Superintendent and Secretary of the Infirmary to all meetings of the House Committee and the Board of Management and can take part in all discussions on any subject and can vote. As a matter of fact they do not interfere as a rule with any subject with which they have no concern, such as finance, etc., to which I have already alluded as being purely lay in nature. They are there more to explain and, if necessary, to advise on points which I think all the members present would consider to be proper for them to deal with, and I do not know of any case in which the explanation or the advice has been resented.

As I have said, this system has worked in the most perfect way at the Manchester Royal Infirmary. At other hospitals, although the staff are represented on the Board, they are not allowed to vote. This is a point which does not seem to me to be of any great importance; the thing that matters is the actual presence of members of the staff with permission to explain and advise. At other hospitals the whole staff sit on the Board. If the staff is small in number and the lay members preponderatingly large in their average attendance I do not see any great objection, but otherwise I do, for if there is about an equality in numbers the public might think the staff was managing the hospital, and this is an idea which it is advisable should not exist. Another objection is that if the whole staff are allowed to attend, then there may be occasions when none is present, each depending too much on the others; for what is everybody's and anybody's work is nobody's work, whereas a few elected representative members of the staff have put upon them the duty of regular attendance. And it is very necessary that this attendance should be regular, for by experience I know how often at the end of some ordinary meeting, just as members are rising, some question is asked or some letter is read which at once fixes the members to their seats and keeps them there for another hour's discussion, and frequently such matters arise which involve and require medical advice and explanation.

I should like before closing to mention one comparatively small matter, but which is important. At some hospitals instead of or in addition to members of the active staff sitting on the Board a member of the retired staff is present. This is, to my mind, undesirable, and especially so if he is the only medical member present, members of the active staff not being admitted. It is undesirable, because members of the retired staff have a tendency to lose touch with or to lose sympathy with the work of younger men, and the advice they may give to the lay members of the Board may be diametrically opposed to the views of the active staff, and their presence on the Board may become a hindrance to the progressive work of the hospital.

To summarise, I would suggest that in every hospital the staff should be represented on the Board of Management by some or all of its active members; that these members, if elected as representatives (which is the method I prefer), should be elected annually, should in no case serve for longer than two years in succession, and should be few in number so that the lay members are always predominant. To those hospitals who have not yet tried this arrangement I would say, "Try it as soon as possible; I have seen the good result."

### The Discussion.

Mr. J. I. Loewy, Jewish Hospital, Manchester:

I represent the only Jewish hospital in the British Empire. We have already adopted the system described by Dr. Reynolds, and have found it to work very well indeed. Three members of the committee are elected by the Medical Board, and we have found it very useful to have them so that we could deal with complaints straight away and without any fuss. We have also adopted representative features which perhaps are not to be found in other hospitals. We have sixty collectors who go out every Sunday collecting, and these young ladies and gentlemen have one representative on the Board. The Ladies' Committee are also represented. We have found this system to answer very well indeed. We are not afraid of the doctors giving an opinion on financial matters, because it can do us no harm and may do us good. (Laughter.) Under our system the Committee is represented on the Medical Board. Our President goes there sometimes, and we have found this to be of the very greatest advantage. I see no reason why laymen should not attend the meetings of the Medical Board. There are matters coming up from time to time when our presence would be very useful, though we would not interfere with purely medical affairs.

Sir Henry Burdett:

With reference to the suggestion of the last speaker that a layman should regularly attend the meetings of the Medical Board, it seems to me that though he might go, it would be very much like a man claiming that because he resides in China he should attend the councils of State there. He might attend, but he certainly would not understand the discussions, although he knew no Chinese. I think, therefore, it would be a lamentable waste of time. (Laughter.) This question is a very important one. And why? Because to-day we have every one of these systems in force in some hospital under the voluntary system. We have hospitals where the medical men are absolutely excluded from taking part in the management. After the paper you have heard, that may seem to be a very serious and wrong thing. But it is based upon a sound principle. The principle is that the Medical Board and the medical staff are a paramount necessity to the hospital. They occupy, and must occupy, a position of immense responsibility which cannot but command respect. The view therefore of those who uphold the policy of their exclusion from direct representation on boards of management is that the medical staff ought, like the House of Lords, to have its own Parliament and its own meetings, and that it should speak in a collective capacity through its resolutions conveyed by the chairman of the Board for the time being, and that in that way all matters affecting the medical well-being of the hospital and the medical staff are best represented. This system certainly upholds the dignity and authority of the medical staff, and on the whole it answers well at times. On the other hand there is the fact that some of the honorary medical officers who are in large practice give absolutely to the cause of the sick, not only their service, but, in the case of the men whose services are in most demand by patients outside, sums which I know amount to hundreds of pounds a year. The consequence of this is seen in the retirement of physicians and surgeons who have made a name for themselves from the honorary posts they have held at hospitals, because they feel as men of honour that the claims of their own patients and the public are so great that they cannot do justice to the claims of the hospital as well. The point I want to get home is that the system referred to fails in practice, because it sometimes interferes with the spirit of unity which I believe to be the bed-rock on which efficiency in voluntary hospital administration rests. Some men feel that they are so not quite represented as they should be. Things are done which the staff does not like, and the consequence is that the system in practice as a whole is not, I consider, the best. The system which the reader of the paper has described as being in force at the Manchester Royal Infirmary is undoubtedly on the whole the best, because it provides reasonable precautions against abuse. You do not have men elected year after year to represent the staff under his system. The regulation whereby none of the representatives serves more than two years and whereby the medical staff have only four representatives on the Board of Management is one that I believe is bound, under our voluntary system, to work best, and it is one which I hope will generally prevail throughout the country.

Dr. G. R. Livingstone, Dumfries Royal Infirmary:

In a small hospital like ours the arrangement you have in Manchester would not really suit. We have two surgeons and two physicians on the staff. They are general practitioners, all engaged in wide country practices, and it would be absolutely impossible for any one of them to attend the Board meetings. What we felt was that we must have some means of communication between the board of directors and the staff, and we formed a medical committee. The whole of our staff are members of that committee. We appoint a chairman and secretary, and we have certain standing orders. Any communications we wish to make to the Board we make formally through our secretary, and when the Board wish to communicate with us they do so through our secretary. For small hospitals I think this is the ideal way of working matters. It works very well with us. (Applause.)

Mr. J. Smethurst, Warrington Dispensary:

In Warrington, with a hospital of eighty beds, we carry out Dr. Reynolds' scheme in its entirety. We find it works very well. The Board of Management consists of fifteen lay members and eight of the honorary staff. From these we appoint a House Committee to look after the house management and the financial side . Then the Medical Board have their own meetings. They send to the House Committee, meeting every week, a representative. That representative is elected on the House Committee every two years, essentially in an advisory capacity. In the event of our having complaints as regards patients and treatment—and we all know they are often made, very often without any cause—we have tried the very satisfactory plan of having joint meetings of our Medical Board and the House Committee. This has worked splendidly for us, and given the greatest possible satisfaction.

Mr. C. W. Thies, Royal Free Hospital, London:

The evolution and development of the Manchester Royal Infirmary runs very much on the same lines as that of the institution with which I have had the honour to be connected. At the Royal Free Hospital, for many years after I was appointed secretary the relations between the lay committee and the medical staff were not at all of the best character. From time to time I suggested to the chairman that matters might be improved by giving the medical staff direct representation on the Board. The first time I made the suggestion it was repulsed at once. But fortunately we secured the services of a very admirable chairman, who saw at a glance that the only way to bring things to a better state was to give the medical staff due representation on the Board, and they were allowed to have two representatives. The medical staff hold their own meetings and send up representations, which receive consideration. With that arrangement in force for the last twelve or thirteen years, the relations between the medical staff and the lay committee have been of the most admirable character. There was another case in London where the medical committee were kept out of the management until things came to such a pass that outside influence had to be brought to bear. The final result was that the medical committee got their way and got their representation, and I believe the relations of the two sides at the hospital are now just as happy as at the Royal Infirmary in Manchester and the Royal Free Hospital in London. These instances bear out the contention of Dr. Reynolds that the medical committee should have, not a preponderating influence, but full opportunity of impressing their view. Almost every question that comes before any lay committee affects the patients through the medical staff. (Hear, hear.)

Dr. T. B. Rhodes, North Staffordshire Hospital:

Our system is a combination of the two suggestions brought before the Conference, and we find it works extremely well. On the General Committee the Medical Board, through the senior honoraries, have members in their own right. That Committee meets once a quarter. But on the House Committee there is direct representation from the Medical Board through their Chairman as such. If there are any special sub-committees formed—as, for instance, a Rules and By-laws Sub-Committee—the Medical Board are asked to appoint representatives to act as their voice.

Dr. F. H. Westmacott, St. John's Hospital for Ear and Eye:

I think the rule should be that in every hospital the Chairman of the Medical Board, holding office for not more than two years, should be the representative on the lay board, and if the size of the medical staff and of the lay board necessitate the regular attendance of at least one member of the medical staff at the meetings, one might send more than one medical representative.

It is necessary also not to fall into the error of at least one hospital—a hospital which had a representation of the whole of its medical staff, both honorary and paid, on the Board of Management. The Board was represented at a Conference of different hospitals, which passed certain resolutions on a matter of policy. When the representatives returned to their Board of Management and asked them to consider the matter, the Medical Board was present in force and over-ruled the resolution, reversed the policy which had been agreed on, and passed a resolution that no further representatives should be sent to the Conference. Such a thing is deplorable in the interests of the hospital concerned.

Mr. H. J. Collins, House Governor General Hospital, Birmingham:

In Birmingham our General Board consists of forty-five members, and, nine being the number of representatives of the medical staff, there is no possibility of that staff outvoting the lay members. But in order that there may be no difficulties with the staff, the arrangement is that the Board of Management, which meets monthly, and at which the minutes of all the committees are read, appoints a House, Finance, and other Committees. On the House Committee a physician and surgeon appointed by the medical staff annually are appointed for two years in alternate years, so that there is always one there who has been on the year before. On the question whether the lay board should be represented on the Medical Committee, I think that that would be impracticable. In Birmingham we have a different principle. We have a buffer as between the medical staff and the Board of Management, and as for the last eighteen years I have been that buffer those present will realise that there is at any rate a good wedge between the two sides. (Laughter.) Many misunderstandings have been averted by the fact that when discussions take place at the Medical Committee, consisting of the whole medical staff, honorary as well as paid, any question discussed at the House Committee, or lay Board of Management, is explained by the House Governor to the medical staff.

Dr. C. H. Melland, Ancoats Hospital, Manchester:

We have had experience of the difficulties resulting from over-representation of the medical staff on the lay committee at the hospital I am attached to at present. Until a recent date the Board of Management was constantly at variance with the Medical Board. But since a member of the Medical Board has represented them on the Board of Management matters have smoothed down in a remarkable and unexpected way.

Dr. Reynolds (in reply):

Dr. Livingstone, of Dumfries, said the staff at his institution would have no time to attend the meetings of the general board. But from the method he described of sending communications backwards and forwards, and the meetings which have to be held to put things straight, as between the medical staff and the Board, it seems to me they are wasting a considerable amount of time, and I would suggest that even in the smaller hospitals the arrangement I have described should be tried.

This concluded the discussion, and the Conference passed to the next and final paper,

### "THE METHODS OF DISTRIBUTION OF HOSPITAL SATURDAY AND SUNDAY FUNDS."

This paper was by Mr. Chas. Hopkinson, member of the Board of the Manchester Infirmary. It was as follows:—

There are two theories of their duty upon which the Managing or Distributing Committees of such Funds may proceed in those places where the funds collected have to be distributed amongst several hospitals:

1. To interpret the wishes of the donors.
2. To apply the funds to produce the maximum amount of effect in relief of pain and disease.

The results of working on either theory may very well be the same.

Considering first the wishes of the donors, obviously there can be no direct evidence available for aggregating and averaging the way in which the individuals who have contributed would divide their separate contributions amongst the hospitals entitled to share. If, however, the donors are regarded as average persons one can classify them into groups and form some idea as to what their wishes would be.

The first group may be defined as the Church and Chapel-goers who put something into the plate or box because such is their habit regardless of the particular object of the collection. They have no definable wishes as to its distribution and are negligible in estimating the aggregate wish.

A second group, probably a small one, includes those who, having subscribed directly to the hospitals they feel most interested in or regard as most important to the community, wish to assist the hospitals they have not directly helped. It is reasonable to suppose that the individuals of this group would wish their contributions distributed amongst the other hospitals in proportion to their importance, which may be measured by the amount of work done. Assuming that the direct contributions of this class are in proportion to the work done by the hospitals, the aggregate desire of the group for division would be also in proportion to the work done by each hospital, including those specially subscribed to. The assumption is probably not quite sound—it is more likely that the subscription lists of the various hospitals represent the proportions of the direct gifts of this class more nearly than the figures of work done. If the two assumptions were combined a very near guess at the truth would, I think, be arrived at.

A third group, also a small one (but one which I know to include certainly at least one individual), would include those who give with a definite view to help all the hospitals, having given separately special subscriptions in proportion to their desire to benefit particular hospitals over and above their general gift.

The fourth, and probably by far the largest group, would include those who do not give directly to any particular hospital but wish to assist hospital work generally in proportion to their capacity to do so.

It is difficult to infer from known facts how these would wish their contributions divided. The interpretation of human nature requires a touch of genius to which I, for one, cannot aspire, but the desire to get as much as possible for any expenditure is sufficiently general to give me some confidence in suggesting that the average donor would desire efficiency and economy of management to be taken into account in dividing up his contribution, that he would prefer, if possible, that his contribution should suffice to provide for an inpatient for X + 1 days rather than for X days only, or for treating X + 1 out-patients rather than X.

The relative poverty of the hospitals would appeal to some who might desire that the extravagance and consequent poverty of one hospital as compared with another, that the lethargy of one hospital as compared with the energy of another in obtaining funds, should be favourable elements in considering distribution—the class, in fact, who would prefer to give a shilling to a beggar in rags on the way to the workhouse rather than to a decent workman to prevent him pawning his tools. The amount of work done is a factor which must naturally appeal to all. One cannot assess these various feelings and aggregate and average them into any reliable estimate. There is, however, in each hospital's accounts one reliable measure of its place in the public mind, and the need of helping it. The donors of individual subscriptions are probably affected by just the same motives as are the members of this fourth group, and it would seem that the average desire would be met by dividing the funds in proportion to the subscription lists of the various hospitals. To adopt such a method of division would have a very stimulating effect on the various hospitals in their efforts to make their utility known and recognised in their subscription lists.

If then, committees are to distribute in accordance with the probable wishes of the donors, they must certainly take into account:—

Efficiency and economy of management,
Amount of work done,
The individual subscription lists, and possibly also, the poverty as measured by the annual deficiency in the accounts.

If, on the other hand, they base their distribution on producing the maximum useful effect, their conclusions can be based on definite facts ascertainable from the statistics and accounts of the hospital.

The ideal system for carrying out this method is that of King Edward's Fund in London, where inspection is made of the hospitals under consideration for a grant by competent lay and medical inspectors, and all the hospitals receiving grants are compelled to keep their accounts on a recognised system facilitating comparison. The grants are used as a powerful stimulus for securing:

Efficiency and economy of management,
Sufficiency of equipment,
Prevention of overlapping and concentration of effort, and succouring poverty-stricken hospitals, whilst the effort is made to improve their financial position.

Thus the distribution of the Fund is made effective, not only by its direct help but by its powerful stimulus to work on right lines, and to make the other funds of the hospital produce greater results. Unfortunately, other towns have neither the same financial nor personal power available as is applied to King Edward's Fund.

To judge the medical and surgical efficiency of a hospital is not practicable to a lay committee—some idea in the University cities may, perhaps, be gathered from inquiry amongst general practitioners. They continue an interest in their poorer and more difficult cases which they have recommended to the charity for treatment, and have a fairly good idea how a hospital is going on. In small towns where the hospital work is mainly done by the general practitioners, no such criticism is available. The economy of work can be measured by the statistics of work done, and the accounts of expenditure. It is very desirable that all hospitals receiving grants should adopt the uniform system of accounts so as to facilitate those comparisons which detect waste.

Between hospitals doing one class of work only comparisons are easy, but when you want to compare an out-patient dispensary with a hospital for in-patients, difficulties arise.

This association might very well remove this initial difficulty by declaring, after suitable consideration, a standard annual average cost per bed occupied by a child in hospital, a convalescent in a Convalescent Home, a medical patient, an operation patient, or grouping these two together to state what would be a fair allowance per operation extra to the average bed, and a standard average cost for each out-patient accident case, each ordinary out-patient case, each home patient (this class will, I suppose, disappear when the proposed insurance system is in full work).

I suggest as tentative figures, and as inviting criticism, the following scale:—

| | | £ | s. | d. |
|---|---|---|---|---|
| Child's bed occupied per annum (18s. weekly) | | 46 | 16 | 0 |
| Convalescent bed occupied per annum (18s. weekly) ... ... ... ... ... ... | | 46 | 16 | 0 |
| In-patient bed occupied per annum (25s. weekly) | | 65 | 0 | 0 |
| Operations involving anæsthetics ... ... | each | 1 | 0 | 0 |
| Out-patient cases, including medicine ... | ,, | 0 | 2 | 6 |
| Out-patient accident cases ... ... ... | ,, | 0 | 2 | 0 |
| Home patients ... ... ... ... ... | ,, | 0 | 2 | 0 |

I suggest, then, that the amount of work should be taken as the basis of distribution, and the cost of that work at standard rates for working out percentages, so that no advantage should be gained by extravagance, and if economy is to be further stimulated a percentage might be deducted from all grants where the standard costs were exceeded. I suggest further that the grant to any hospital be limited to the amount of its true deficiency for the year. In case modification is desired a minor part, say, not exceeding one-third, might be distributed in proportion to the subscription lists, or the deficiencies of the hospitals as shown by the differences between the aggregate income and the standard cost for the work done.

I am sorry to say that in Manchester and Salford you will not find a good example in the method of distribution, and the effect is, I think, reflected in the amount collected, which indicates that the public do not regard the fund with that enthusiasm we hospitals managers desire.

Let me confess our shortcomings, which I have endeavoured unsuccessfully to amend.

We do not encourage efficiency, we encourage extravagance in management by taking gross expenditure as a factor instead of the work done, or a standard expenditure based on the work done.

We pay no attention to equipment.

We make no attempt to prevent overlapping.

In short, we make no effort to apply the funds collected to produce the maximum effect for good. We limit our assistance to the principal hospital which does fully one-third of the whole voluntary hospital work of the community here to one-fourth of the fund—a direct invitation to its managers to reduce the amount of work done by closing some of its wards, and to refrain from increasing its facilities for serving the sick and injured poor.

The system of distribution adopted is so artificial and unsound that I fear I may have difficulty in making it quite clear to you.

The gross expenditure is taken of each hospital, and from that gross is deducted the aggregate receipts from invested funds, direct collections, profits from private nurses, payments for or by patients, students' fees, and so arrive at a nominal deficiency in proportion to which the funds are distributed, limiting the amount paid to one-fourth of the fund to any one hospital.

You will observe that no regard is paid to income from subscriptions or legacies or large donations, so that it is quite conceivable that a hospital should be saving money, and yet receiving substantial grants. On this system this old infirmary was at one time receiving grants and investing money annually, now in this new, enlarged, and entirely modern hospital it shows, according to the Sunday Fund accounts, a deficiency of more than one-third of the whole deficiency of the hospitals assisted, and yet is only allowed to receive one-quarter of the fund. Is it possible to have a stronger condemnation of the method?

---

### The Discussion.

Mr. L. W. Glenton-Kerr, Great Northern Hospital, London:

It is quite clear that unless the three factors of economy, efficiency, and work are considered by collecting bodies in allotting their funds, injustice must be done to the institutions which participate in the grants. I would suggest as a remedy in the distribution in Manchester and Salford, that some closer connection should be instituted between the participating institutions and the collecting and dividing body. If that body could have the defects brought clearly before them, they would take early means of remedying them.

Mr. C. Swinglehurst, Vice-Chairman of the Hospital Saturday and Convalescent Home Fund, Manchester:

For a number of years various arguments have been brought before us for changing the method of distributing the funds, and year after year we have come to the conclusion that the method already adopted was the best for distributing the money. Our method is the method Mr. Hopkinson has described. We have had it pointed out to us that certain hospitals re-enter their patients at the end of every month as fresh patients, so that a hospital adopting that method appears to have done more work at the end of the year than an institution maintaining its patients for three or six months. Mr. Hopkinson's paper might be sent to our institution, and I can promise you it will be discussed, and we will do anything we can to bring about a better administration of the funds at our disposal.

Sir Henry Burdett:

The remark of the last speaker with regard to the Hospital Saturday Fund is reassuring, because he shows they have a knowledge of methods in making up the returns of patients which, to say the least, are undesirable, and have been properly denounced in the past as dishonest. No committee in charge of a hospital ought to permit any figures to go out unless they can stand the strictest test and audit, and can be proved to be true. (Hear, hear.) Another point is that knowledge can only be acquired by investigation, and in this regard I think that in a large community like Manchester it is desirable to have one distribution committee for all funds raised through organisations like the Hospital Saturday and Sunday; and I do not see why it should not be possible to have a good number of visitors appointed to visit in couples, one being a member of the medical profession who has a knowledge of hospitals and one a layman of position, with a good business knowledge, as is done in London in connection with the King's Fund. These visitors should make a periodical visit of inspection to every hospital participating, and make a careful report on the condition and circumstances of every particular charity. Thus, if any question arises as to a particular hospital, it can be referred to the visitors, who can visit the hospital, and as men known by their public position they can go thoroughly into the matter. In that way the distributing committee can command a knowledge of the facts not attainable in any other way. The question of the distribution of your Hospital Saturday and Sunday Fund is of vast importance, especially in view of the Insurance Bill. At present we want a prophet as of old to go out and see the clergy and make them understand that Hospital Sunday is their day for personal service. (Hear, hear.) It is Samaritan Day for the churches, and the men who freely do the work and give their service are the clergy of all denominations. I remember what a great day Hospital Sunday was some forty years ago. The streets of Birmingham were quite a sight on that day. Men and women who never entered a place of worship during the rest of the year went on Hospital Sunday. Whole families went. It was a day that offered all creeds an opportunity of uniting to give a helping hand to the sick. That is the spirit which, if we could get it back, would help the churches and the Fund. It is a very bad business for any parson not to make the most of Hospital Sunday, because he fears that if he puts his strength into it it may affect his other collections.

The wider you throw the net of contribution, the larger the field of contribution, the better it is in every way for the individual church and all its collections. The ministers want to increase their area of givers, and when they do that they will find that the actual amount of the collections for every object in a year will go up and not go down.

Mr. Hopkinson briefly replied on the discussion, and his reply closed the session and the proceedings of the Conference.

